# Case of Fungus from a Wound in the Ear

**Published:** 1800

**Authors:** Henry Fryer

**Affiliations:** Surgeon at Stamford.


XI. *Caſe of Fungus from a Wound in the Ear. By the ſame.*

A Boy, between fifteen and ſixteen years old, was thrown from a horſe on the 31ſt of Auguſt, 1797, and his left ear was torn ſo much acroſs, rather below the middle, that I put a ſtitch through it; the lobe and outer edge of the ear were alſo excoriated. The wound went on very well, and in a few days ſeemed nearly healed, the ſtitch having been taken out, and adheſive plaſter only uſed. He neglected to come to me for ſome days, thinking it well, in which time he had a blow upon it, and when he came again, a fungus had begun to riſe upon the whole of the injured part, which continued to increaſe, in ſpite of every common method, as blue vitriol, lunar cauftic, &c. I one day pared it down, which produced violent hæmorrhage, fo that I was forced to apply lint, dipped in oil of turpentine to ftop it. It continued to increafe in this way for fome weeks, till it was as large as half an orange. I then determined to try the effect of arfenic and antimony,* fprinkling fome of it upon lint, and applying it over the fungus; this adhered fo clofely that it was not moveable for a day or two. I then found it had acted on the tumor, without having caufed much pain; it was, therefore, continued occafionally, till the whole of the fungus was deftroyed, and dropped off; which it did by degrees, having turned black, but without much fmell, and left a clean healthy fore, which healed without any further trouble; but the cure was not effected in lefs than fifteen weeks from the time of the accident.

There was fome forenefs on the neck juft below the ear, from the difcharge running upon it, when the arfenical preparation was ufed.

* Vide Pharmac. Chirurg. p. 6. 3d edit.

